# Evaluation of *In Vitro* Anticancer Activity of *Ocimum Basilicum, Alhagi Maurorum, Calendula Officinalis* and Their Parasite *Cuscuta Campestris*

**DOI:** 10.1371/journal.pone.0116049

**Published:** 2014-12-30

**Authors:** Mandana Behbahani

**Affiliations:** Department of Biotechnology, Faculty of Advanced Sciences and Technologies, University of Isfahan, Isfahan 81746-73441, Islamic Republic of Iran; Taipei Medical University, Taiwan

## Abstract

The present investigation was carried out to study the relationship between presence of cytotoxic compounds in *Ocimum basilicum, Alhagi maurorum, Calendula officinalis* and their parasite *Cuscuta campestris*. The cytotoxic activity of the pure compounds was performed by MTT assay against breast cancer cell lines (MCF-7 and MDA-MB-231) and normal breast cell line (MCF 10A). The induction of apoptosis was measured by the expression levels of p53, bcl-2, bax and caspase-3 genes using quantitative Real Time PCR. Three active fractions were detected by nuclear magnetic resonance as lutein, lupeol and eugenol, respectively, in *C. officinalis*, *A. maurorum* and *O. basilicum*. These compounds and their epoxidized forms were also detected in their parasite *C. campestris*. The cytotoxic activity of lutein epoxide, lupeol epoxide and eugenol epoxide was significantly more than lutein, lupeol and eugenol. The mRNA expression level of p53, caspase-3 and bax genes were increased in both cancer cells treated with all pure compounds. However, bcl-2 gene expression decreased in treated breast cancer cells. In conclusion, all the data indicated that the epoxide forms of lupeol, lutein and eugenol are potential drug candidates for inducing apoptosis in human breast cancer cells.

## Introduction

Breast cancer is one of the most common diseases among women worldwide and the majority of cases have been reported in Asian countries over the past two decades [1]. Currently, many studies have been carried out worldwide to isolate the active novel compounds from plants for cancer treatment. Several secondary metabolites including alkaloids, polyphenols, flavonoids and triterpenes were purified from medicinal plants [2]. These products cause apoptosis which is modulated by direct or indirect modulating expression of some genes such as p53, bcl2 and caspase-3 [3], [4]. However, it is important to develop the natural chemopreventive agents which evaluate cytotoxicity and apoptosis induction in cancer cell lines. Therefore, we became eager to assess Iranian species with respect to their anti-cancer activity and determine their active constituents. The genus *Cuscuta* (Convolvulaceae) also known as dodder is an obligate stem parasite with a worldwide distribution. *Cuscuta campestris* is the most common species in the genus *Cuscuta* and grown on wide range of host species [5]. In recent decades, through extracting natural compounds from *cuscuta* specious a number of compounds such as cuscutin, amarbelin, β-sitosterol, stigmasterol, kaempferol, dulcitol, myricetin, quercetin, coumarin and oleanolic acid have been isolated [6], [7]. *C. campestris* has been proved to have analgesic, antipyretic, anti-inflammatory and anti-cancer properties on different hosts [8]. Some studies have reported that some secondary metabolites transferred from host to the parasite and responsible for medical properties of parasite [9], [10]. Three Iranian common hosts for *C. Campestris* are *Alhagi maurorum*, *Calendula officinalis* and *Ocimum basilicum*. Medicinal activity of these plants has been also subjected by several investigations [11]–[13]. So far, cytotoxic activity of *C.campestris* and the relationship with its hosts has not been investigated yet. Hence, the present study is focused to evaluate anticancer potential of pure compounds isolated from *A. maurorum*, *C. officinalis*, *O. basilicum* and their parasite *C. campestris* against breast cancer cell lines (MCF7 and MDA-MB231) and human normal breast cancer cell line (MCF 10A).

## Materials and Methods

### Ethics statement

No ethics statement was necessary for this study. The study was only carried out *in vitro* on an immortalized cell line. None of these species is an endangered or protected species and did not need a permit.

### Plant material

The aerial parts of *O. basilicum, A. maurorum, C. officinalis* and their parasite *C. campestris* were collected from university of Isfahan herbarium, Iran, in Feb 2011. The specimen was identified in University of Isfahan herbarium, Iran. One kg of each sample was carefully dried in a well-ventilated dark room and powdered. Finally, the dried powders were obtained.

### Extraction and isolation of compounds

Methanol extracts of the powdered aerial parts of *O. basilicum*, *A. maurorum*, *C. officinalis* and their parasite *C. campestris* (50 g) were prepared. The extraction was done three times at room temperature (25–28°C) by the maceration method (3×24 h). The crude methanol extract of each sample was concentrated by a rotary evaporator (Steroglass, Italy) and freeze dried (Zirbus, Germany). Silica-gel column fractionation chromatography was separately performed with 5 g of each methanol extract and eluted with hexan: acetone: methanol (1∶0∶0 to 0∶0∶10, v/v). The following fractions have been obtained from these three plants and their parasite. The anticancer activity of all fractions were verified by MTT assay.

Fractions 1–17 (0.31, 0.20, 0.21, 0.25, 0.29, 0.22, 0.31, 0.21, 0.25, 0.36, 0.20, 0.21, 0.35, 0.19, 0.20, 0.18 and 0.20 g) from *O. basilicum* and fractions 1–23 from its parasite *C. campestris* (0.37, 0.18, 0.14, 0.35, 0.28, 0.36, 0.17, 0.21, 0.18, 0.23, 0.22, 0.28, 0.30, 0.21, 0.21, 0.14, 0.22, 0.30, 0.20, 0.17, 0.18, 0.17 and 0.27 g) were obtained. Fraction 13 from *O. basilicum* and fractions 17 and 23 from *C. campestris* were the most active fractions.

Fractions 1–25 (0.25, 0.15, 0.18, 0.22, 0.20, 0.13, 0.21, 0.14, 0.25, 0.24, 0.21, 0.19, 0.18, 0.2, 0.21, 0.21, 0.16, 0.2, 0.18, 0.18, 0.21, 0.15, 0.20, 0.15 and 0.20 g) from *A. maurorum* and fractions 1–27 from its parasite *C. campestris* (0.19, 0.15, 0.21, 0.2, 0.18, 0.20, 0.21, 0.20, 0.15, 0.19, 0.3, 0.32, 0.25, 0.20, 0.2, 0.18, 0.18, 0.19, 0.22, 0.20, 0.25, 0.16, 0.16, 0.25, 0.21, 0.20, and 0.20 g) were achieved. Fraction 15 from *A. maurorum* and fractions 16 and 21 from its parasite *C. campestris* were the most active fractions.

Fractions 1–26 (0.20, 0.21, 0.16, 0.20, 0.22, 0.2, 0.18, 0.17, 0.15, 0.19, 0.21, 0.21, 0.21, 0.21, 0.22, 0.21, 0.21, 0.15, 0.20, 0.19, 0.23, 0.24, 0.22, 0.2, 0.21, and 0.20 g) from *C. officinalis* and fractions 1–24 from its parasite *C. campestris* (0.15, 0.19, 0.21, 0.23, 0.24, 0.23, 0.21, 0.23, 0.24, 0.22, 0.2, 0.21, 0.21, 0.22, 0.24, 0.21, and 0.20 g) were obtained. Fraction 19 of *C. officinalis* and fractions 14 and 17 of *C.campestris* were the most active fractions. The active fractions were detected with Nuclear Magnetic Resonance (NMR).

### Instrumental analysis

NMR screening was used to identify trace compounds in each fraction. ^1^H NMR and ^1^C NMR data were recorded on Bruker 400 MHz spectrometer by use of CDCl_3_ and DMSO as residual solvent with chemical shifts expressed in parts per million (ppm).

### Culture medium and cell lines

The cell line MCF 10A, MCF-7 and MDA-MB-231 were acquired from National Cell Bank of Pasture Institute, Tehran, Iran. Cell lines were cultured in Dulbecco's Modified Eagle Medium (DMEM) complemented with 10% heat-inactivated Fetal Bovine Serum (FBS), 100 U/ml penicillin and 100 µg ml^−^ streptomycin and 5 mM L^−^ Glutamine. The cell lines were grown at 37°C in a humidified atmosphere containing 5% CO_2_. All reagents and cell culture media were purchased from the Gibco Company (Germany).

### Cytotoxicity assay

The cytotoxic activity of active fractions isolated from *O. basilicum, A.maurorum, C. officinalis and* their parasite *C. campestris* on cultured cells were measured using MTT assay [14]. The cells were grown in 96-well plates at a density of 5×10^4^ cells per well. After incubation for 24 h, the cells were treated with different concentrations of samples and incubated for 48 h. Later, the MTT solution (25 µl of 5 mg/ml, Roche) was added to each well, and the plate was re-incubated for an additional 4 h. Finally, the medium was removed and 100 µl of DMSO was added to solubilize the formed formazan crystals. The amount of formazan crystal was determined by measuring the absorbance at 492 nm using a microplate spectrophotometer (Awareness Technology Inc., stat fax 2100). The 50% cytotoxic concentration (CC50) of all pure compounds was calculated. All assays were done in triplicate.

### Determination of the expression levels of apoptosis-regulatory genes

Expression levels of four widely established apoptotic-related mRNAs, p53, bcl-2, bax and caspase-3 were analyzed using Real Time PCR assay as described previously [15], [16]. MCF-7 and MDA-MB-231 cells were treated with all pure compounds at 1/4 of CC50 values for 6 and 12 h periods. Total cellular RNA was isolated from the untreated and treated cells using the Tri-Pure Isolation Reagent (Roche, USA), according to the manufacturer's instructions.

### Quantitative real-time polymerase chain reaction assay for p53, bcl-2, bax and caspase-3

Real Time PCR was performed to quantify the amount of mRNA in untreated and treated cells. A PCR reaction mixture of 50 µl containing 5 µl of dH2O, 25 µL of Taq Man Universal PCR MasterMix, 5 µl of primer forward, 5 µl primer reverse, 5 µl FAM- TAMRA probes, 0.5 µl of reverse transcriptase, 2 µl random hexmer and 2 µl of purified RNA was used. Five pairs of primers were separately used: four pairs to amplify the p53, bcl2, bax and caspase-3 genes, the other pair for the endogenous control gene, gapdh. The primers and probes have been shown in Table 1. Real-time PCR was carried out on Corbett Cycler. Cycling conditions were as follows: initial reverse transcription at 55°C for 45 min, 1 cycle denaturation of 95°C with 10 min hold, followed by 40 cycles of 95°C with 15 s hold, annealing temperature at 60°C (p53, bcl2, bax, caspase-3 and gapdh) with a 60 s hold. A negative control was included in each run to access specificity of primers and possible contamination. Primers and probes were synthesized by Metabion Company (Germany).

**Table 1:** The primers and probe sequences used in real-time PCR assay.

Gene	Sequence
**p53**	Forward: 5′-AGAGTCTATAGGCCCACCCC-3′
	Reverse: 5′-GCTCGACGCTAGGATCTGAC-3′
	Probe: 5′-(FAM) TTGGGCAGTGCTCGCT-MGB-3
**bcl-2**	Forward: 5′-TTCGATCAGGAAGGCTAGAGTT-3′
	Reverse: 5′-TCGGTCTCCTAAAAGCAG GC-3′
	Probe: 5′-(FAM)CCCAGAGCATCAGGCCGCCAC(TAMRA)-3′
**gapdh**	Forward: 5′-CATGGGGAAGGTGAAGGTCGA-3′
	Reverse: 5′-TTGGCTCCCCCCTGCAAATGAG-3′
	Probe: 5′-(JOE)CCGACTCTTGCCCTTCGAC(TAMRA)-3′
**caspase-3**	Forward: 5′-TGCGCTGCTCTGCCTTCT-3′
	Reverse: 5′-CCATGGGTAGCAGCTCCTTC-3′
	Probe: 5′-FAM-AGCTTCTTCATTTGTGTGCTCCGCTTTCA(TAMRA)-3′
**bax**	Forward: 5′ -CATGTTTTCTGACGGCAACTTC -3′
	Reverse: 5′- AGGGCCTTGAGCACCAGTTT-3′
	Probe: 5′-(FAM) CCGGGTTGTCGCCCTTTTCTACTTTG(TAMRA)-3′

### Western blot analysis

The expression levels of p53, caspase-3, bcl-2 and bax proteins in MCF-7 and MDA-MB-231 cells were assessed by western blot method as described by Fido et al. [17]. Both cells (5×106 cells/ml) were treated with lutein epoxide, lupeol epoxide, eugenol epoxide, lutein, lupeol and eugenol at 1/4 of CC50 values for 48 h at 37°C. Cells were lysed with 10 µl of lyses buffer (120 mmol/L Tris-HCl, 2 mmol/L N-ethylmaleimide, 2 mmol/L phenylmethyl sulfonylfluoride, 4% sodium dodecylsulfate, 4% dithiothreitol, 20% glycerol, 0.01% bromophenol blue, 2 mol/L urea and 10 mmol/L Na-EDTA at pH = 6.8. Cell lysates were centrifuged at 16000 rpm/min for 20 min at 4°C. 50 µg of each sample was separately resolved by SDS-PAGE and move onto a nitrocellulose membrane overnight at 30 mA. Membranes were blocked with 2% BSA diluted in PBS for 1 h at 37°C. Membranes were incubated with saturating concentrations of primary antibody (anti-p53; anti-caspase-3, anti-bcl2, anti-bax) for 2 h under gentle agitation at room temperature. The blots were washed three times and incubated with horse reddish peroxdase-conjugated anti-mouse IgG antibody for 1 h at 37°C. Then the signal was detected with an enhanced chemiluminescence detection kit (Roche, Mannheim, Germany). Diaminobenzidine reagent was used to develop the immunoblots.

### Statistical analysis

The data of three independent experiments are presented as mean±SD. The CC50 values were calculated by Microsoft Excel 2003. The student's unpaired t-test was used to evaluate significance between the test sample and solvent control. One-way analysis of variance (ANOVA) followed by a Dunnett post hoc test was performed to evaluate significance differences among different groups. P value <0.05 was shown to be statistically significant.

## Results

### NMR Analysis

The most active fraction obtained from *O*. *basilicum* was fraction 13, which analyzed by NMR experiment as eugenol. Fractions 17 and 23 were also isolated from its parasite *C.campestris* and determined as eugenol and eugenol epoxide. Fraction 15 of *A*. *maurorum* and fractions 16 and 21 of *C.campestris* were analyzed as lupeol, lupeol and lupeol epoxide respectively. Fraction 19 of *C. officinalis* and fractions 14 and 17 of *C.campestris* were analyzed as lutein, lutein and lutein epoxide.

### Eugenol epoxide

^1^HNMR (CDCl_3_, 400 MHz): δ 6.80 (d, 1H, J = 7.9 Hz), 6.75 (d, 1H, J = 1.8 Hz), 6.64 (dd, 1H, J = 1.8, 7.9 Hz), 5.60 (s, 1H), 3.85 (s, 3H), 3.02 (m, 1H), 2.80 (dd, 1H, *J*_1_ = 5.5, *J*_2_ = 14.7 Hz), 2.73 (dd, 1H, *J*_1_ = 2.4, *J*_2_ = 4.9 Hz), 2.68 (dd, 1H, *J*_1_ = 5.6, *J*_2_ = 14.8 Hz), 2.50 (dd, 1H, *J*_1_ = 4.9, *J*_2_ = 2.4 Hz).

^13^CNMR (CDCl_3_, 100 MHz) δ 147.46, 145.28, 133.14, 122.15, 115.22, 112.78, 56.24, 54.76, 48.32, 41.34.

### Eugenol

^1^HNMR (CDCl_3_, 400 MHz):δ 7.12 (s, 1H), 6.78 (d, 2H, *J* = 7.2 z), 6.32–6.42 (m, 2H), 5.45–5.65 (m, 2H), 3.85 (s, 3H), 3.26 (m, 2H)

^13^CNMR (CDCl_3_, 100 MHz) δ 147.23, 145.01, 138.23, 133.14, 122.15, 117.01, 114.52, 112.36, 56.24, 41.03.

### Lupeol

^1^H NMR (CDCl_3_, 400 MHz): δ 4.84 (d, 1H, *J* = 2.4 Hz), 4.62 (d, 1H, *J* = 2.4 Hz), 3.18 (1H, m), 2.42 (m, 1H), 1.80 (m, 1H), 1.71 (t, 1H, *J* = 5.6Hz), 1.60 (s, 3H), 1.57 (m, 2H), 1.40 (m, 2H), 1.36 (m, 2H), 1.20 (m, 1H), 1.03 (m, 1H), 0.99 (s, 3H), 0.97 (s, 3H), 0.95 (s, 3H), 0.83 (s, 3H), 0.79 (s, 3H), 0.75 (s, 3H).

^13^C NMR (CDCl_3_, 100 MHz): δ 149.12, 111.25, 80.24, 57.15, 51.63, 49.44, 48.16 41.23, 41.06, 41.02, 40.62, 39.85, 38.94, 38.23, 37.36, 36.21, 34.56, 32.04, 29.21, 28.01, 27.15, 24.36, 22.13, 19.85, 19.01, 18.45, 17.14, 16.78, 15.27, 14.42.

FT-IR [KBr, ν (cm^−1^)]: 3359, 3075, 2948, 2868, 1651, 1461

### Lupeol epoxide

^1^HNMR (CDCl_3_, 400 MHz): δ 3.23 (1H, m), 2.59 (d, 1H, *J* = 4.8 Hz), 2.42 (m, 1H), 2.28 (d, 1H, *J* = 4.8 Hz), 1.80 (m, 1H), 1.70 (t, 1H, *J* = 5.6Hz), 1.63 (s, 3H), 1.57 (m, 2H), 1.40 (m, 2H), 1.36 (m, 2H), 1.20 (m, 1H), 1.03 (m, 1H), 0.99 (s, 3H), 0.97 (s, 3H), 0.95 (s, 3H), 0.83 (s, 3H), 0.79 (s, 3H), 0.75 (s, 3H).

^13^CNMR (CDCl_3_, 100 MHz): δ 79.42, 64.32, 58.14, 53.21, 50.36, 48.65, 48.01, 42.47, 42.01, 41.74, 40.59, 39.84, 38.94, 38.25, 37.34, 36.21, 34.56, 32.12, 30.15, 28.16, 28.02, 24.03, 22.13, 19.86, 19.03, 18.56, 17.17, 16.78, 15.27, 14.42.

### Lutein

^1^HNMR (DMSO, 400 MHz): δ 6.21–6.76 (m, 13H), 5.52 (m, 1H), 3.82 (m. 1H), 3.41 (m, 1H), 3.68 (s, 1H), 2.95 (s, 1H), 2.71 (d, 1H, *J* = 9.2 Hz), 1.58–2.36 (m, 6H), 1.69 (s, 12H), 1.71 (s, 6H), 1.01 (s, 6H), 0.85 (s, 6H).

^13^CNMR (DMSO, 100 MHz): δ 138.11, 137.82, 137.65, 136.81, 136.50, 135.06, 131.24, 131.01, 130.65, 130.01, 129.85, 128.06, 126.66, 125.02, 68.35, 65.01, 57.32, 48.02, 43.91, 42.98, 37.65, 32.34, 28.01, 26.62, 24.31, 21.93, 14.35, 13.78

### Lutein epoxide

^1^HNMR (DMSO, 400 MHz): δ 6.18–6.73 (m, 13H), 5.61 (m, 1H), 3.61 (s, 2H), 3.55 (m, 1H), 3.21 (m, 1H), 2.43 (d, 1H, *J* = 9.2 Hz), 1.89 (s, 12H), 1.55–1.85 (m, 6H), 1.29 (s, 6H), 0.92 (s, 12H).

^13^CNMR (DMSO, 100 MHz): δ 137.64, 136.95, 136.14, 135.24, 133.61, 132.58, 132.34, 130.45, 130.23, 129.41, 128.02, 71.02, 68.32, 75.05, 67.23, 65.12, 55.15, 52.18, 47.24, 43.34, 41.27, 35.03, 29.87, 28.31, 27.62, 24.32, 20.14, 13.65, 13.46

### Cytotoxicy assay

**Figure 1:**
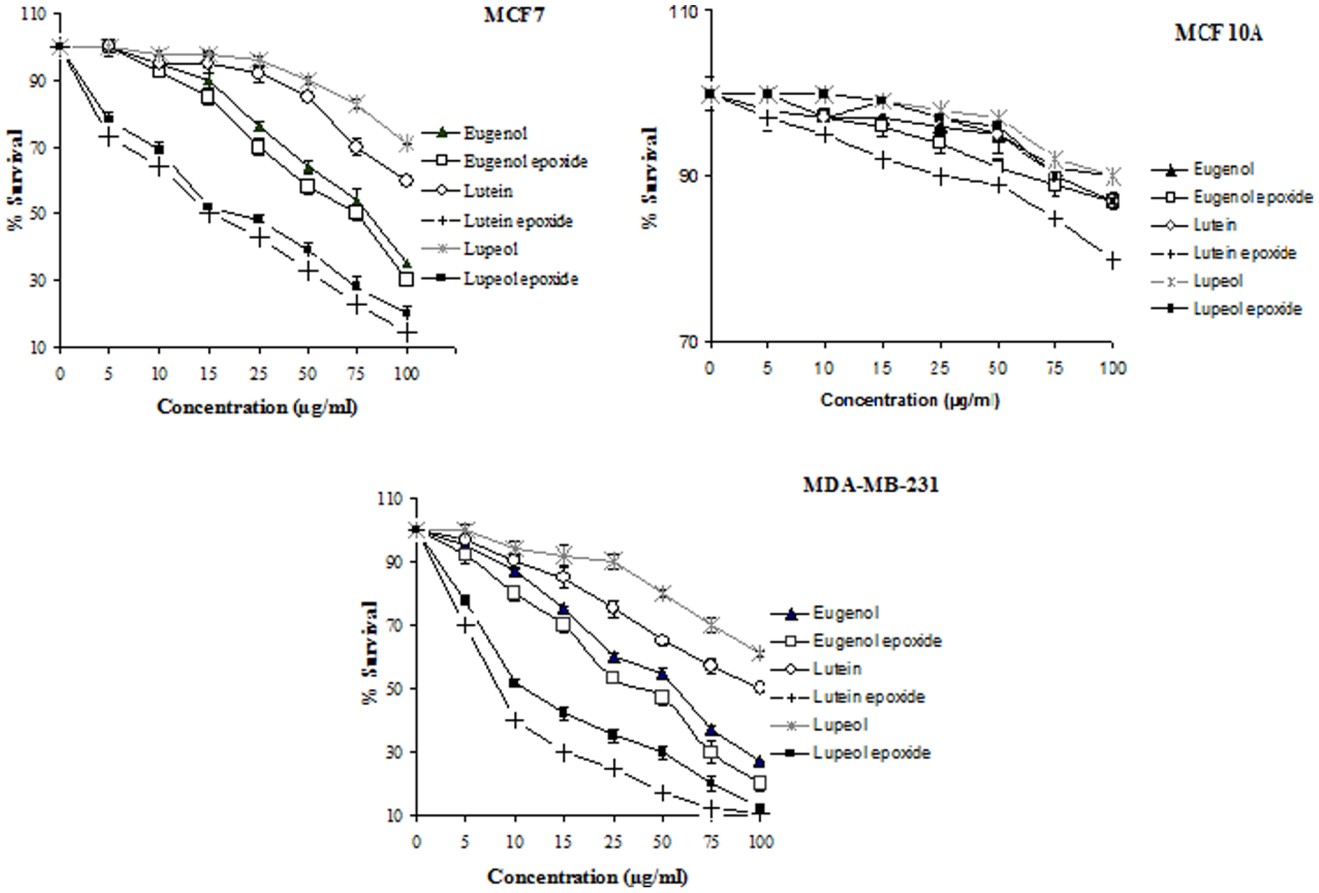
Cytotoxic activity of lutein epoxide, lupeol epoxide, eugenol epoxide, lutein, lupeol and eugenol against MCF7, MDA-MB231 and MCF 10A cell lines.

Different concentrations of lutein, lupeol, eugenol, lutein epoxide, lupeol epoxide, eugenol epoxide were tested for cytoxicity against MCF7, MDA-MB-231 and MCF 10A cells. These compounds were tested under comparable conditions at different concentrations (5, 10, 15, 25, 50, 75 and 100 µg/ml). All pure compounds showed dose-dependent cytotoxic activity on MCF7 and MDA-MB-231 cell lines. The 1/4 of CC50 values of lutein, lupeol, eugenol, lutein epoxide, lupeol epoxide and eugenol epoxide were, respectively, >100, >100, 84, 15, 17.5, 75 µg/ml for MCF7 and 100, >100, 55, 7.5, 11.5, 48 µg/ml for MDA-MB-231. As a result, cytotoxic activity of lupeol, lutein, lupeol epoxide and lutein epoxide against MDA-MB cancer cell line was rather than MCF7 cancer cell line. The cytotoxic activity of lupeol, lutein and eugenol were significantly lower than epoxide forms of these compounds. The results also showed that cytotoxic activity of these extracts on MCF7 and MDA-MB cells were significantly more than MCF 10A cells (Fig 1).

### Expression level of apoptosis-related genes

**Figure 2:**
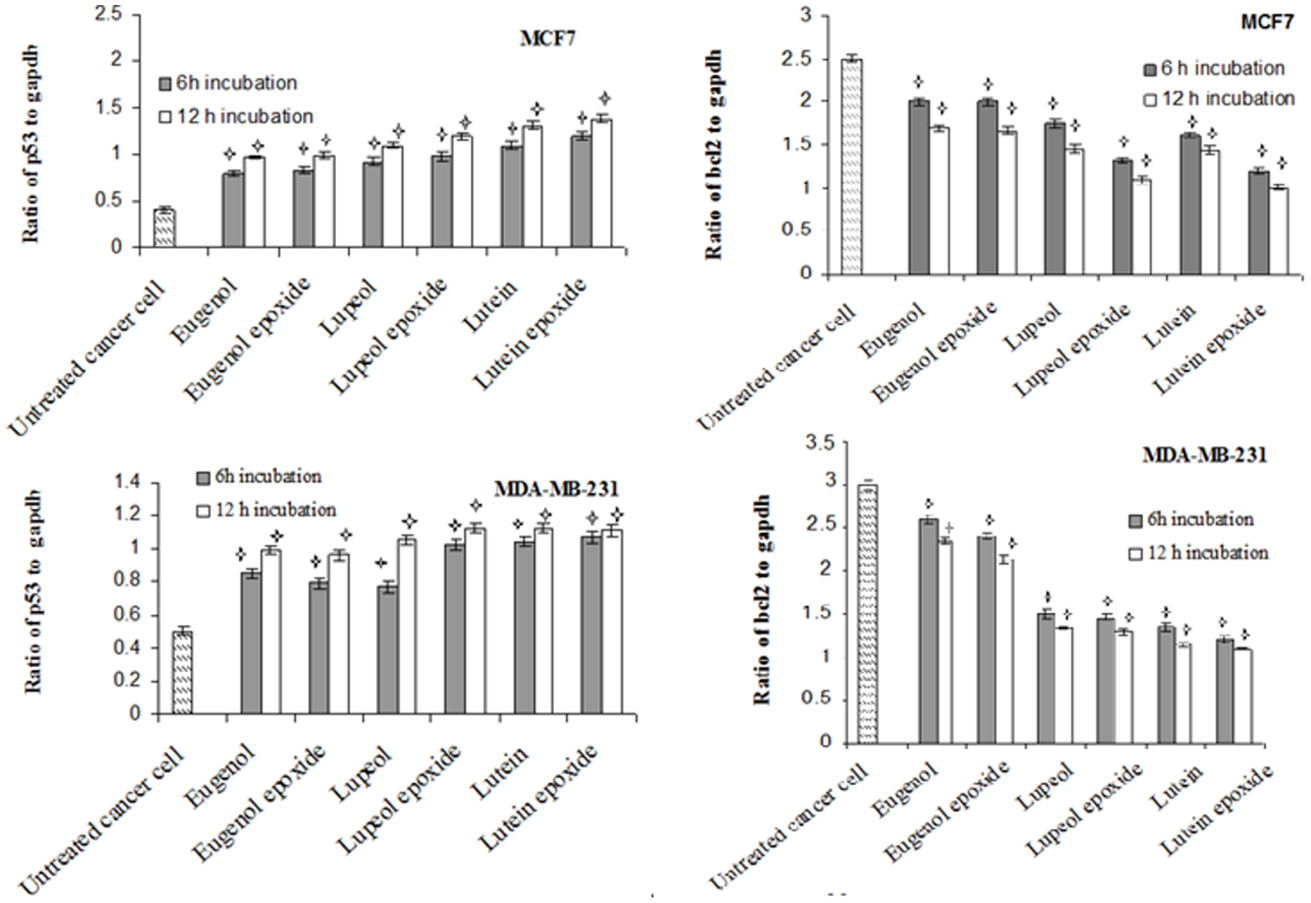
Time dependency effects of p53 and bcl-2 mRNA levels in human breast cancer cell line, MCF7, incubated with *lutein, lupeol, eugenol, lutein epoxide, lupeol epoxide, eugenol* epoxide at 1/4 of CC50 values for 6 h and 12 h incubation. gapdh was used as an endogenous control gene. The stars indicate that the data are significantly different (p<0.05) from the untreated control.

**Figure 3:**
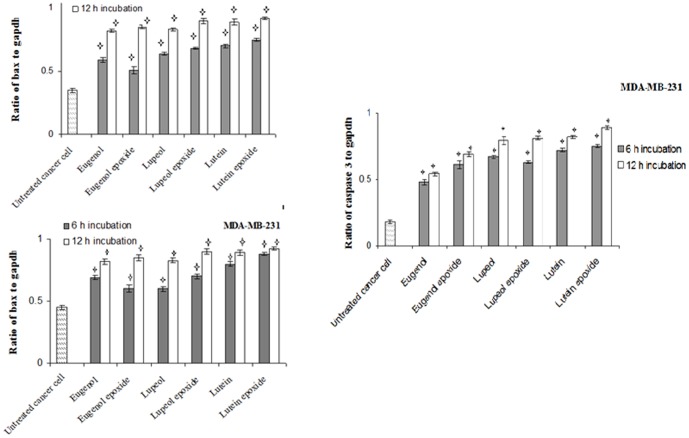
Time dependency effects of caspase 3 and bax mRNA levels in human breast cancer cell line, MCF7 and MDA-MB 231 incubated with *lutein, lupeol, eugenol, lutein epoxide, lupeol epoxide, eugenol* epoxide at 1/4 of CC50 values for 6 h and 12 h incubation. gapdh was used as an endogenous control gene. The stars indicate that the data are significantly different (p<0.05) from the untreated control.

The expression levels of apoptosis-related genes in MCF7 and MDA-MB 231 cells which induced by with 6 separate pure compounds at 1/4 of CC50 values were determined. The mRNA levels of p53, caspase3, bax and bcl-2 were evaluated by Real time PCR. Fig. 2 showed that the expression level of p53 and bcl2, respectively, increased and decreased in both cells treated with lupeol, lupeol epoxide, lutein, lutein epoxide, eugenol and eugenol epoxide for 6 and 12 h incubation compared to untreated cells. The expression level of p53 and bcl2 in both breast cancer cells treated with these compounds was time dependent. Fig. 3 indicated that the relative expression of bax was increased in cells treated with all pure compounds for 6 and 12 h incubation compared to untreated cells. The relative expression of caspase-3 in MDA-MB-231 cancer cells treated with these extracts was also increased as time-dependent to reach the maximum level at 12 h after stimulation. The absence of caspase-3 in MCF-7 cell leads to lack of any gene expression in treated and untreated cells (data not shown).

### Western blot analysis

**Figure 4:**
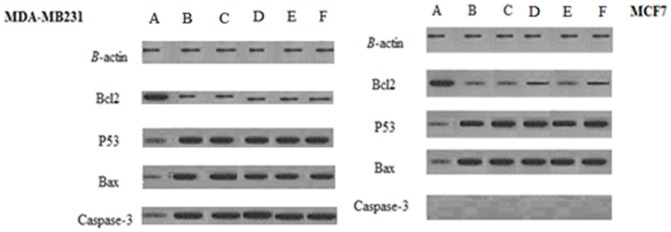
Western blot analysis of MDA-MB-231 and MCF7 treated with lutein (A), lupeol (B), eugenol (C), lutein epoxide (D), lupeol epoxide (E) and eugenol epoxide (F). Western blot analysis was performed with monoclonal antibodies to human bcl-2, p53 and bax. β-actin was used as loading control.

The expression level of bcl2, bax and p53 proteins in MCF7 and MDA-MB23 cells treated with lutein, lupeol, eugenol, lutein epoxide, lupeol epoxide, eugenol epoxide along with β-actin as an internal control are shown by western blotting analysis. p53 and bax genes expressed, respectively, 53-kda and 21-kda proteins on western blotting. MDA and MCF7 cells also encode a 32-kDa and 24-kda proteins whose association with caspase-3 and bcl2 proteins. As shown in Fig. 4, Western blot analysis showed the increase in band intensity of p53 and bax proteins in MCF7 and MDA cells when compared to the internal control β-actin. caspase-3 was also increased in MDA cells compared to β-actin. The absence of caspase-3 gene in MCF-7 cell leads to lack of any caspase-3 protein in treated and untreated cells. bcl2 protein was decreased in cancer cells treated by all compounds at 1/4 of CC50 values.

## Discussion

Medicinal plants have been used widely in traditional medicine for cancer treatment. In this study, lutein epoxide, lupeol epoxide and eugenol epoxide isolated from *C. campestris* were considered as potential anticancer compounds. Lutein and lutein epoxide are plant cartenoids which is widely distributed in leaves and fruits of many plants such as tangerine, olive fruits, peas and pumpkins [18], [19]. Antioxidant and cytotoxic activity of lutein and oxidized lutein in Hela cells have been report previously [20]. Lupeol and lupeol epoxide are naturally occurring triterpenoids which found in many plants, including olive, mango, crataeva and strawberry [21]. However, lupeol and its derivatives have also been reported to possess a wide spectrum of medicinal properties such as anticancer, antibacterial and antifungi properties [22]–[24]. Eugenol and its derivatives have been identified in various plants, such as basil, cinnamon, lemon balm and clove [25]. In the present study, the active compounds of *lutein*, *lupeol* and *eugenol* were observed, respectively, in *C. officinalis*, *A. maurorum* and *O. basilicum*. The epoxide forms of these compounds were also detected in their parasite *C. campestris*. The mRNA expression level of p53, caspase-3 and bax genes were increased and bcl-2 gene expression decreased in breast cancer cells treated with these six compounds. The bcl-2 proteins are able to inhibit programmed cell death. So the expression of bcl-2 corresponds with the status of p53 in cells. Bax is another pro-apoptotic protein, which increases apoptosis in cells [26]. The result of this study demonstrated that p53 is involved in apoptosis which induced by lutein, lupeol, eugenol, lutein epoxide, lupeol epoxide and eugenol epoxide. There would be decreased expression of bcl-2 and increased expression of bax proteins in treated cells compared with non–treated cells. Importantly, the ratio of bax/bcl-2 protein expression after treatment was dose-dependently increased which indicated the susceptibility of MCF-7 and MDA-MB-231 cells toward apoptosis. This study concluded that some anticancer compounds transferred from host to the parasite *C. campestris* and change to epoxide forms.

## Conclusions

The results of detection of bioactive compounds signified that eugenol epoxide, lutein epoxide and lupeol epoxide constituted the most active fractions in the crude methanol extract of *C. campestris* and displayed the cytotoxic effects on breast cancer cell.
